# Effect of the molar ratio of (Ni^2+^ and Fe^3+^) on the magnetic, optical and antibacterial properties of ternary metal oxide CdO–NiO–Fe_2_O_3_ nanocomposites

**DOI:** 10.1038/s41598-023-36262-6

**Published:** 2023-06-03

**Authors:** Asma A. A. Al-Mushki, Abdullah A. A. Ahmed, A. M. Abdulwahab, Salem A. S. Qaid, Nasser S. Alzayed, Mohammed Shahabuddin, Jameel M. A. Abduljalil, Fuad A. A. Saad

**Affiliations:** 1 Department of Physics, Faculty of Applied Science, Thamar University, 87246 Dhamar, Yemen; 2 Department of Physics and Astronomy, College of Science, King Saud University, PO Box 2455, Riyadh, 11451 Saudi Arabia; 3 Department of Biology, Faculty of Applied Science, Thamar University, 87246 Dhamar, Yemen

**Keywords:** Biophysics, Materials science, Nanoscience and technology

## Abstract

In this work, the effect of the molar ratio of (Ni^2+^ and Fe^3+^) on the properties of CdO–NiO–Fe_2_O_3_ nanocomposites was investigated. The synthesis of CdO–NiO–Fe_2_O_3_ nanocomposites was carried out by self-combustion. XRD, UV–Vis, PL and VSM were used to describe the physical properties of the materials. The results showed significant progress in structural and optical properties supporting antibacterial activity. For all samples, the particle size decreased from 28.96 to 24.95 nm with increasing Ni^2+^ content and decreasing Fe^3+^ content, as shown by the XRD pattern, which also shows the crystal structure of cubic CdO, cubic NiO, and cubic γ-Fe_2_O_3_ spinel. The Ni^2+^ and Fe^3+^ contents in the CdO–NiO–Fe_2_O_3_ nanocomposites have also been shown to enhance the ferromagnetic properties. Due to the significant coupling between Fe_2_O_3_ and NiO, the coercivity H_c_ values of the samples increase from 66.4 to 266 Oe. The potential of the nanocomposites for antibacterial activity was investigated against Gram-positive (*Staphylococcus aureus*) and Gram-negative (*Pseudomonas aeruginosa*, *Escherichia coli*, and *Moraxella catarrhalis*) bacteria. Comparison of *P. aeruginosa* with *E. coli*, *S. aureus* and *M. catarrhalis* showed that it has a stronger antibacterial activity with a ZOI of 25 mm.

## Introduction

The synthesis of nanomaterials is at the heart of the current research field of nanotechnology, which offers a wide range of interesting applications, e.g. in the fields of electrochemistry, biomedicine, catalysis, cosmetics, electronics, optics and optical devices, energy science, mechanics, food technology, healthcare, sensors, textile technology, space technology and pharmaceuticals^1–8^.

CdO is a well-known n-type semiconductor with piezoelectric properties and polycrystalline nature^4,9^. As a result, cadmium oxide nanoparticles (CdO NPs) are extensively used in various applications, including photovoltaic cells, photodiodes, transparent electrodes, gas sensors, infrared detectors, liquid crystal displays, antireflective coatings, and solar cells^10–13^. CdO is an excellent photocatalyst for photocatalytic applications due to its ability to absorb visible light and its high carrier mobility^14,15^. Due to their unusual physiochemical properties, CdO NPs are effective against malaria, bacteria, tuberculosis, and cancer^4,9,16^.

Fe_2_O_3_, an environmentally friendly semiconducting oxide material, is widely used in biomedicine, catalysts, and batteries. Apart from these applications, Fe_2_O_3_ is a promising candidate for a variety of technological applications^17^. Fe_2_O_3_ has shown promise for applications such as drug delivery, organic impurity removal, and MRI imaging^18,19^. Due to its high surface-to-volume ratio, Fe_2_O_3_ with nanometric dimensions exhibits modified properties^20,21^. Due to their superparamagnetic properties, nontoxicity, and biocompatibility, they are becoming increasingly popular. It is promising as catalytic material, absorbent, magnetic recording device, ion exchanger, gas sensor and other applications. Iron oxide is the most stable and environmentally friendly oxide in the world^22–24^.

NiO is one of the most important transition metal oxides with a wide range of properties when reacting with polar surface materials and is used in a variety of applications due to its excellent chemical and thermal stability, antibacterial activity, environmental friendliness and industrial use^25^.

The capabilities of the individual metal oxides have been greatly enhanced by combining them into innovative nanocomposites, opening up new possibilities for applications in photocatalysis, electro- and optoelectronics, and biology^26^.

The synthesis of CdO–NiO–ZnO nanocomposites for photocatalytic and antibacterial properties was discussed by Karthik et al. Together with tested foodborne pathogens, the nanocomposite showed strong antibacterial activity^27^. Karthik et al. have reported CdO–NiO nanocomposites. The composite showed significant antibacterial activity against foodborne pathogens^28^. Tushar et al. reported the antibacterial activity of α-Fe_2_O_3_-ZnO in the core shell^29^. Balamurugan et al. reported the preparation of CdO-Al_2_O_3_-NiO nanocomposites for photocatalytic and magnetic properties. The composite exhibited weak ferrimagnetic assemblies, making it suitable for magnetic applications^30^. Gnanamoorthy et al. have reported rGO/ZnCo_2_O_4_ nanocomposites and x-CuTiAP nanospheres for antimicrobial applications. The nanocomposites showed antimicrobial activity^31,32^.

This work aims to investigate the effects of the conditions for the preparation of CdO-NiO-Fe_2_O_3_ nanocomposites by the self-combustion method on the structural, optical, magnetic and antibacterial activity.

## Experiment

### Materials

Cadmium nitrate tetrahydrate (Cd(NO_3_)_2_·4H_2_O, Scharlau, 99%), nickel nitrate hexahydrate (Ni (NO_3_)_2_·6H_2_O, Fluka, 98%), iron nitrate nonahydrate (III) (Fe (NO_3_)_3_·9H_2_O, Scharlau, extra pure), polyvinyl alcohol cold water soluble ((–CH_2_CHOH–)_n_, HIMEDIA, 99.99%), and deionized water (DW) were used for the present work. The chemical materials were used in this work without further purification.

### Sample preparation

The CdO–NiO–Fe_2_O_3_ nanocomposites were prepared by the self-combustion method^33^. Briefly, dissolve 5 g of PVA in 200 ml of DW, followed by vigorous stirring for 2 h at 50 °C. The PVA solution was obtained as a gel-like and homogeneous solution. This solution was donated by solution A. Different ratios of nickel and iron, while cadmium was kept constant (Table 1), was prepared separately in 3 solutions. At room temperature, the solution was stirred for 10 min to obtain a homogeneous transparent solution. The solutions of Ni nitrate, Fe nitrate and Cd nitrate were mixed with constant stirring for another 10 min at room temperature. The product solutions were mixed with solution A for 20 min with constant stirring. The stirred solutions were placed in the drying oven for 3 h at 80 °C. The crushed products were calcined at 500 °C for 2 h.

**Table 1:** Samples code at various molar ratio of Ni^2+^ and Fe^3+^.

Sample code	Molar ratio of salts (M)			Weight of salts (g) at 100 ml of DW			Molar ratio of PVA (g/ml)
	Cd^2+^	Ni^2+^	Fe^3+^	Cd^2+^	Ni^2+^	Fe^3+^	
CNF1	0.2	0.05	0.15	4.6	1.1	4.6	0.025
CNF2	0.2	0.1	0.1	4.6	2.2	3.03	0.025
CNF3	0.2	0.15	0.05	4.6	3.3	1.52	0.025

### Characterizations

X-ray diffraction (XRD) was used to investigate the structural features of the fabricated samples (XD-2 X-ray diffractometer with Cu Kα (λ = 1.54 at 36 kV and 20 mA, China). A UV–Vis spectrophotometer (SPECORD 200) was used to measure the absorption spectra of the samples in the range of 190–1100 nm at room temperature. A spectrofluorometer (RF-5301PC; Shimadzu) with an excitation wavelength of 325 nm, an excitation and emission gap of 5 nm, an average scanning speed, and high sensitivity was used to record the photoluminescence spectra (PL) of the fabricated samples. The physical property measurement system (PPMS), QUANTUM DESIGN (MODEL6000), was used together with the attached vibrating magnetometer (VSM) to obtain the magnetic hysteresis (M-H) loops. The solvent for the prepared samples used to measure the absorption and photoluminescence spectra was dilute sulfuric acid (H_2_SO_4_).

### Antibacterial activity

The modified Kirby-Bauer disk diffusion test of the European Committee for Antimicrobial Susceptibility Testing was used to investigate the antibacterial activities of CdO–NiO–Fe_2_O_3_ nanocomposites against Gram-positive (*Staphylococcus aureus*) and Gram-negative (*Pseudomonas aeruginosa*, *Escherichia coli*, and *Moraxella catarrhalis*) bacteria^34^. Biochemical assays were used to further verify the identity of the isolates before testing the nanocomposites. The nanocomposites were serially diluted twice from the 75 mg/ml stock solution and suspended in sterile distilled water. The disks were impregnated with four different working dilutions. 450, 225, 112.5, and 56.25 μg/disk were prepared by impregnating a sterile filter paper disk (6 mm diameter) with 12 μl (6 μl on each side) for dilution. The plates were inoculated with swabs to form a uniform bacterial lawn on the agar surface. Using sterile forceps, the plates were positioned on the infected agar surface and incubated for 18–20 h at 37 °C. After completion of the incubation period, the diameters of the inhibition zones were measured to the nearest millimeter. In addition to the disks containing azithromycin as a positive control, a blank disk consisting solely of distilled water was used as a negative control.

## Result and discussion

### XRD analysis

**Figure 1:**
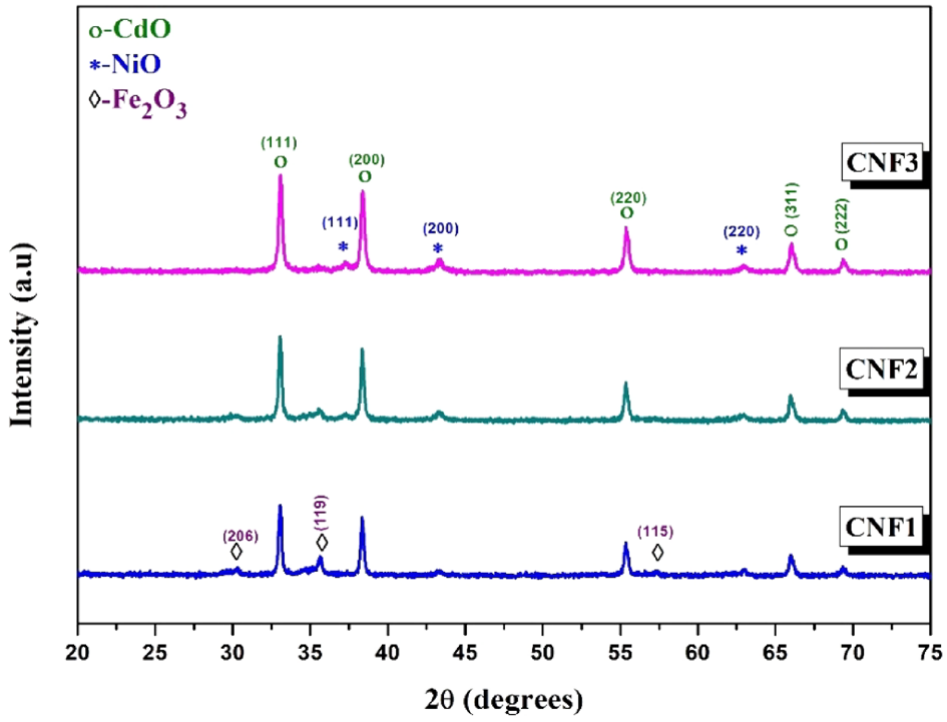
XRD patterns of CdO–NiO–Fe_2_O_3_ nanocomposites at various molar ratio of Ni^2+^ and Fe^3+^. The figure exhibited the crystallization of the oxides an increase as the molar ratio different from CNF1 to CNF3.

The crystal structure of the CdO–NiO–Fe_2_O_3_ nanocomposite was investigated using the XRD technique. In Fig. 1, the XRD patterns of the prepared nanocomposites show only the crystalline phase of CdO, NiO and Fe_2_O_3_ for all samples. The patterns show the face-centered cubic structure of CdO and NiO, while Fe_2_O_3_ exhibits pure maghemite (γ-Fe_2_O_3_ phase with a cubic spinel crystal structure). The CdO patterns at 2θ and its crystal plane at 33°(111), 38.3°(200), 55.34°(220), 65.94°(311), and 69.34°(311) correspond to JCPDS Map No. 00-005-0640^35^. The NiO patterns at 2θ and its crystal plane at 37.18°(111), 43.30°(200), and 63.04°(220) are consistent with JCPDS Map No. 47-1049^36^. The patterns of the γ-Fe_2_O_3_ phases at 2θ and their crystal plane at 30.2°(206), 35.5° (119), and 57.2°(115) are consistent with JCPDS Map No. 00-025-1402^37^. It is clear that the intensity of the peaks of γ-Fe_2_O_3_ decreases with decreasing Fe content, while the intensity of the peaks of NiO increases with increasing Ni content.

The XRD data obtained indicate that an increase in Ni^2+^ and a decrease in Fe^3+^ content causes a change in crystal lattice parameters and a decrease in crystallite size, as indicated in Table 2.

**Table 2:** The Unit cell parameter (*a*), average crystalline size (D), lattice strain (ε) and the dislocation density (δ) of CdO–NiO–Fe_2_O_3_ nanocomposites at various molar ratio of Ni^2+^ and Fe^3+^.

Samples	Unit cell parameter, *a* (nm)			D (nm)	ε × 10^−3^	δ × 10^−3^ (nm^−2^)
	CdO [fcc]	NiO [fcc]	Fe_2_O_3_ [fcc]			
CNF1	0.4697	0.4173	0.834	28.95	4.22	1.19
CNF2	0.4690	0.4176	0.835	26.89	4.54	1.38
CNF3	0.4690	0.4175	0.834	24.94	4.90	1.61

The Scherrer equation^38–43^ is used to calculate the average crystal size of nanocomposites in the crystal plane of CdO (111), which can be given as follows:

1 $$D = \frac{K\lambda }{{\beta \;cos\theta }}$$

where K is the dimensionless form factor (K = 0.9), λ is the X-ray wavelength (= 0.1540 nm), β is the full width at half maximum (FWHM), and θ is the Bragg diffraction angle.

The microstrain (ε) of a nanocrystal is caused by defects in the nanocrystal, such as distortions and imperfections. The microstrain can be calculated using the following equation (ε)^43–45^:

2 $$\varepsilon = \frac{\beta }{{4\;{\text{tan}}\theta }}$$

the dislocation density can be described by the following equation (δ)^39,43,45,46^:

3 $${\updelta } = \frac{1}{{D^{2} }}$$

As seen in Table 2, the particle size decreased from 28.96 to 24.95 nm with increasing Ni^2+^ and decreasing Fe^3+^ content. The decrease in particle size of nanocomposites is attributed to the difference between the ionic radii of Ni (0.074 nm), Cd (0.097 nm) and Fe (0.055 nm)^47^. The dependence of particle size on dislocation density and microstrain. The values of microstrain and dislocation density increase due to the large effect of particle size on the comprehensive stress of the nanocomposite^48^.

### Optical properties

#### Absorption spectra

**Figure 2:**
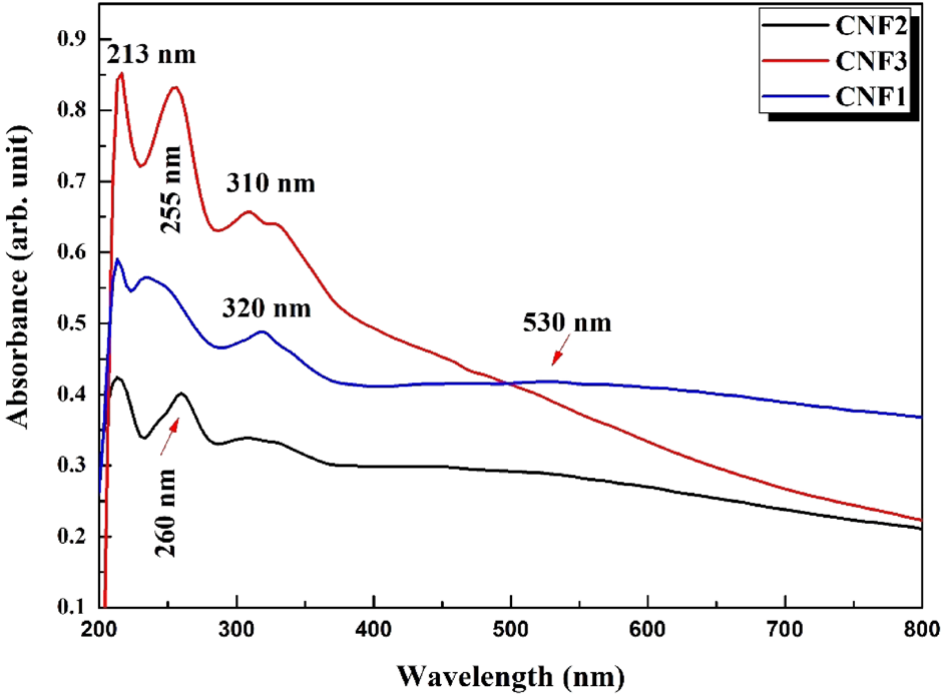
Absorbance spectra of CdO–NiO–Fe_2_O_3_ nanocomposites at various molar ratio of Ni^2+^ and Fe^3+^. The figure showed the absorption peaks for oxides.

The absorption spectra of CdO–NiO–Fe_2_O_3_ nanocomposites at different molar ratios of Ni^2+^ and Fe^3+^ were studied in the wavelength range of (200–800 nm) as shown in Fig. 2. The absorption peaks observed at 213–260 nm are attributed to the absorption band of CdO, while the absorption peaks observed at 310–320 nm are attributed to the absorption band of NiO in the nanocomposite^46^. In special CNF1 samples, a tiny absorption band at 530 nm was observed for Fe_2_O_3_. This absorption band is caused by the absorption of Fe^2+^ and Fe^3+^ ions of iron oxide^49^.

#### Band gap energy (Eg)

**Figure 3:**
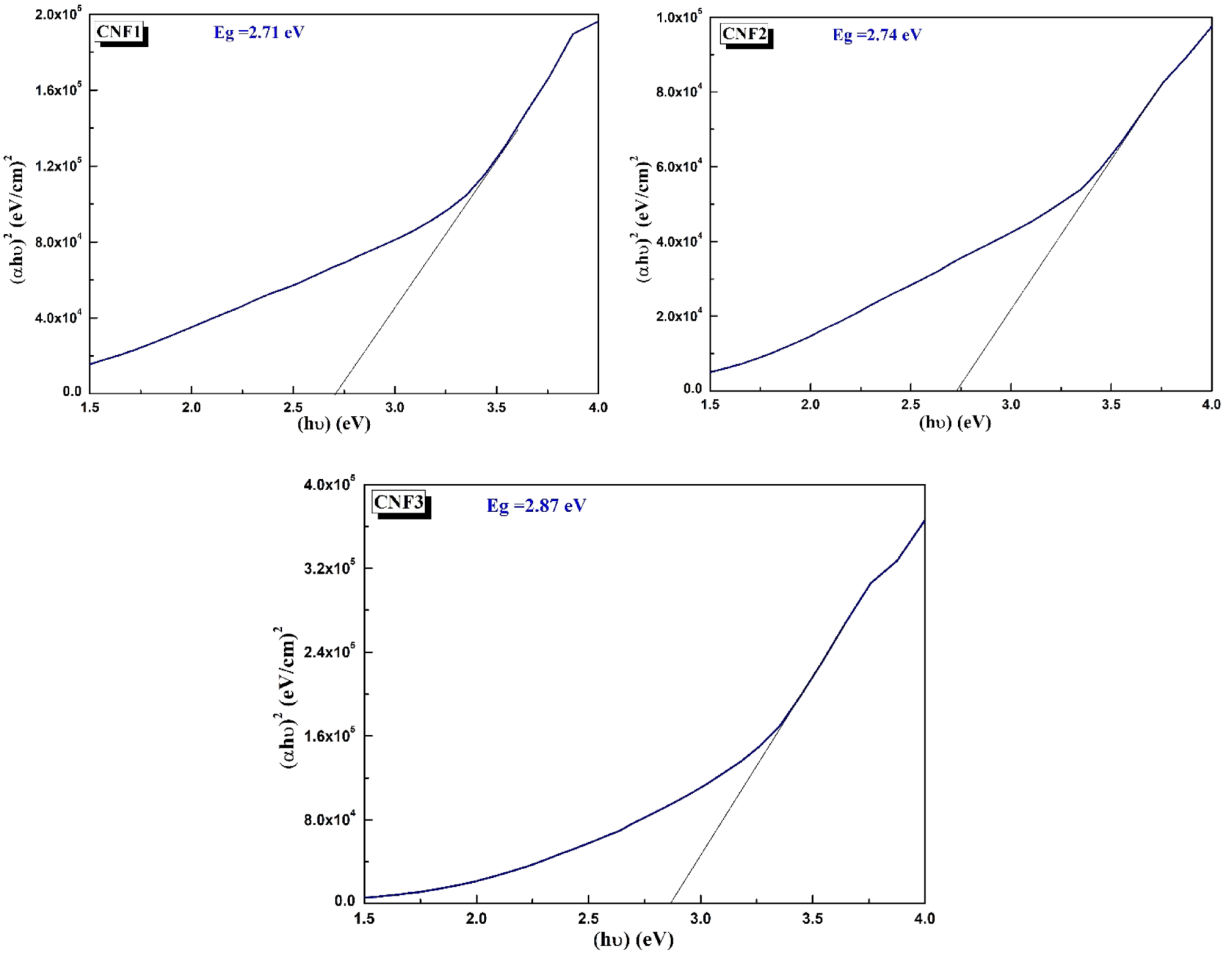
Optical band gap of CdO–NiO–Fe_2_O_3_ nanocomposites at various molar ratio of Ni^2+^ and Fe^3+^.

As seen from Table 3 and Fig. 3, the optical band gap (${E}_{g}$) of the samples is between NiO (3.6 eV)^50^, CdO (2.5 eV)^51,52^ and Fe_2_O_3_ (2 eV)^53^. For the samples, the optical band gap (${E}_{g}$) increased with increasing Ni^2+^ content and with decreasing Fe^3+^ content. The decrease in band gap is related to the grain size. As localized energy states emerge and approach the conduction band, the energy band gap decreases in nanocomposites with a high content of Cd^+2^^46,48^.

**Table 3:** Optical band gap energy measurements of CdO–NiO–Fe_2_O_3_ nanocomposites at various molar ratio of Ni^2+^ and Fe^3+^.

Samples	Energy band gap, *Eg* (eV)
CNF1	2.71
CNF2	2.74
CNF3	2.87

#### PL study

**Figure 4:**
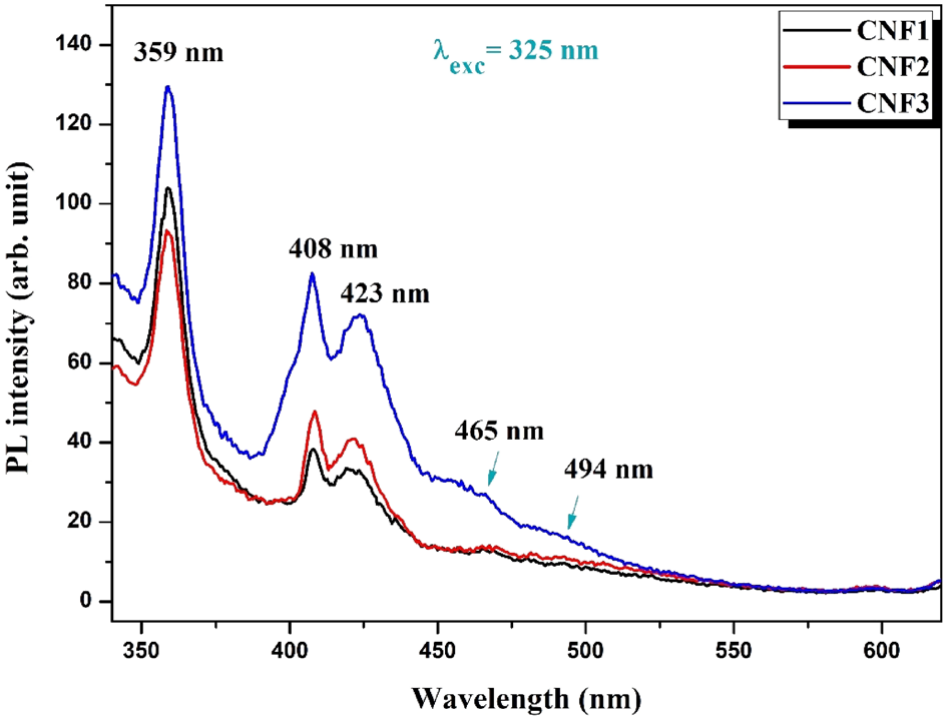
PL spectrum of CdO–NiO–Fe_2_O_3_ nanocomposites at various molar ratio of Ni^2+^ and Fe^3+^.

Figure 4 shows the PL spectra of CdO-NiO-Fe_2_O_3_ nanocomposites at 325 nm and room temperature. The near band edge (NBE) emission of NiO nanoparticles in a nanocomposite matrix was responsible for the observed UV emission peak at 359 nm^54^. Radiative recombination is responsible for the NBE peak in NiO in the exciton-exciton collision process^55^. The trapped electrons migrating into the valence band at the Ni interstitial are thought to be responsible for the strong violet emission peaks at 408 and 423 nm^40^. In CdO–NiO–Fe_2_O_3_ nanocomposites, band gap defects such as oxygen vacancies were responsible for the weak blue emission peaks between 463 and 494 nm^56,57^.

### Magnetic properties

**Figure 5:**
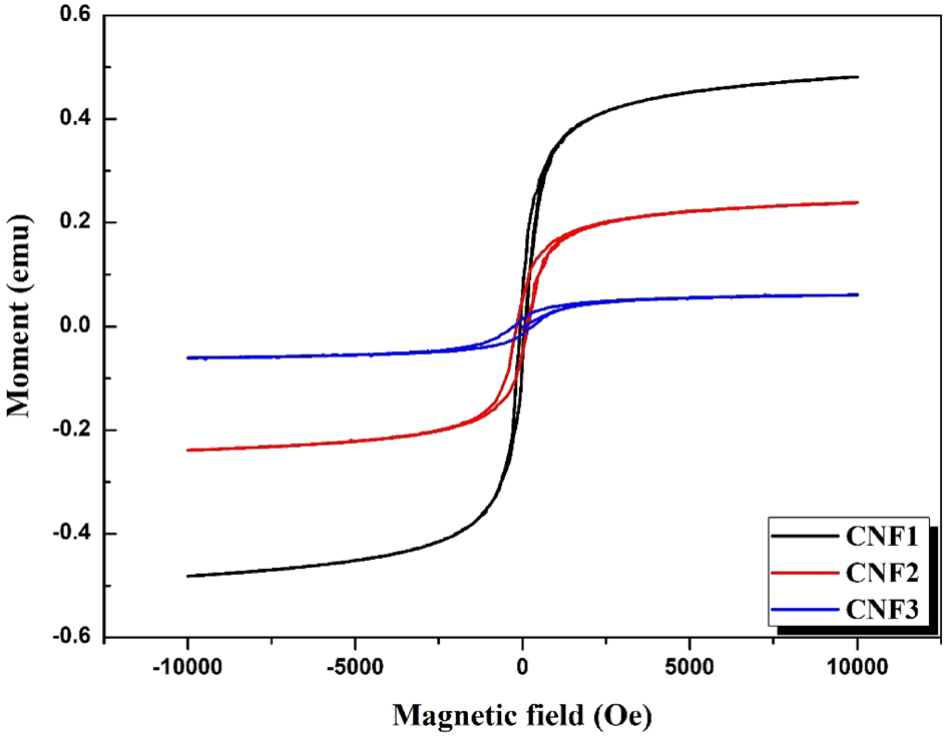
Magnetic hysteresis curves of CdO–NiO–Fe_2_O_3_ nanocomposites at various molar ratio of Ni^2+^ and Fe^3+^. The figure showed variation of the coercivity (${H}_{c}$), and saturation magnetization (${M}_{S}$) as the molar ratio different from CNF1 to CNF3.

VSM was used to analyze the magnetic properties of CdO-NiO-Fe_2_O_3_ nanocomposites at room temperature. Using a magnetic field of 10,000 Oe, magnetic hysteresis measurements were performed on CdO-NiO-Fe_2_O_3_ nanocomposites. As shown in Fig. 5, all samples exhibit ferromagnetic properties, which can be attributed to the presence of Fe_2_O_3_ in all three samples. The CdO-NiO-Fe_2_O_3_ nanocomposites showed weak ferromagnetism as the measured particle size was above the critical value (10 nm)^58,59^. The values of saturation magnetization (M_S_) decrease from 0.482 to 0.060 emu with increasing Ni^2+^ content and decreasing Fe^3+^ content in the samples due to the antiferromagnetic property of NiO and ferromagnetic nature of Fe_2_O_3_^60,61^. The coercivity H_c_ of the samples increases from 66.4 to 266 Oe, which can be attributed to the strong coupling between Fe_2_O_3_ and NiO^62^. It has been shown that the content of Ni^2+^ and Fe^3+^ in the CdO–NiO–Fe_2_O_3_ nanocomposites increases the ferromagnetic properties. The ferromagnetism of the CdO–NiO–Fe_2_O_3_ nanocomposites was increased at room temperature by replacing the nonmagnetic Cd with the magnetic transition metal ions Ni^2+^ and Fe^3+^. Moreover, the ferromagnetism of the nanocomposites increased when oxygen vacancies were formed in them^15,63^. Thus, the causes of the ferromagnetic properties of the metal oxides are the presence of unpaired electron spins arising from surface effects, oxygen/cation vacancies on the surfaces of the samples, and/or the presence of a secondary/impure phase^15,58^. The magnetic parameters (${H}_{c} ,{M}_{r} ,\mathrm{and}{ M}_{S})$ are listed in Table 4.

**Table 4:** Magnetic coercivity (${H}_{c}$), remanent magnetization (${M}_{r}$), and saturation magnetization (${M}_{S}$) of CdO–NiO–Fe_2_O_3_ nanocomposites at various molar ratio of Ni^2+^ and Fe^3+^.

Sample	Coercivity (Oe)	Remanent (emu)	Saturation magnetization (emu)
CNF1	66.4	0.0632	0.482
CNF2	176	0.0511	0.238
CNF3	266	0.0140	0.060

### Antibacterial activity

**Figure 6:**
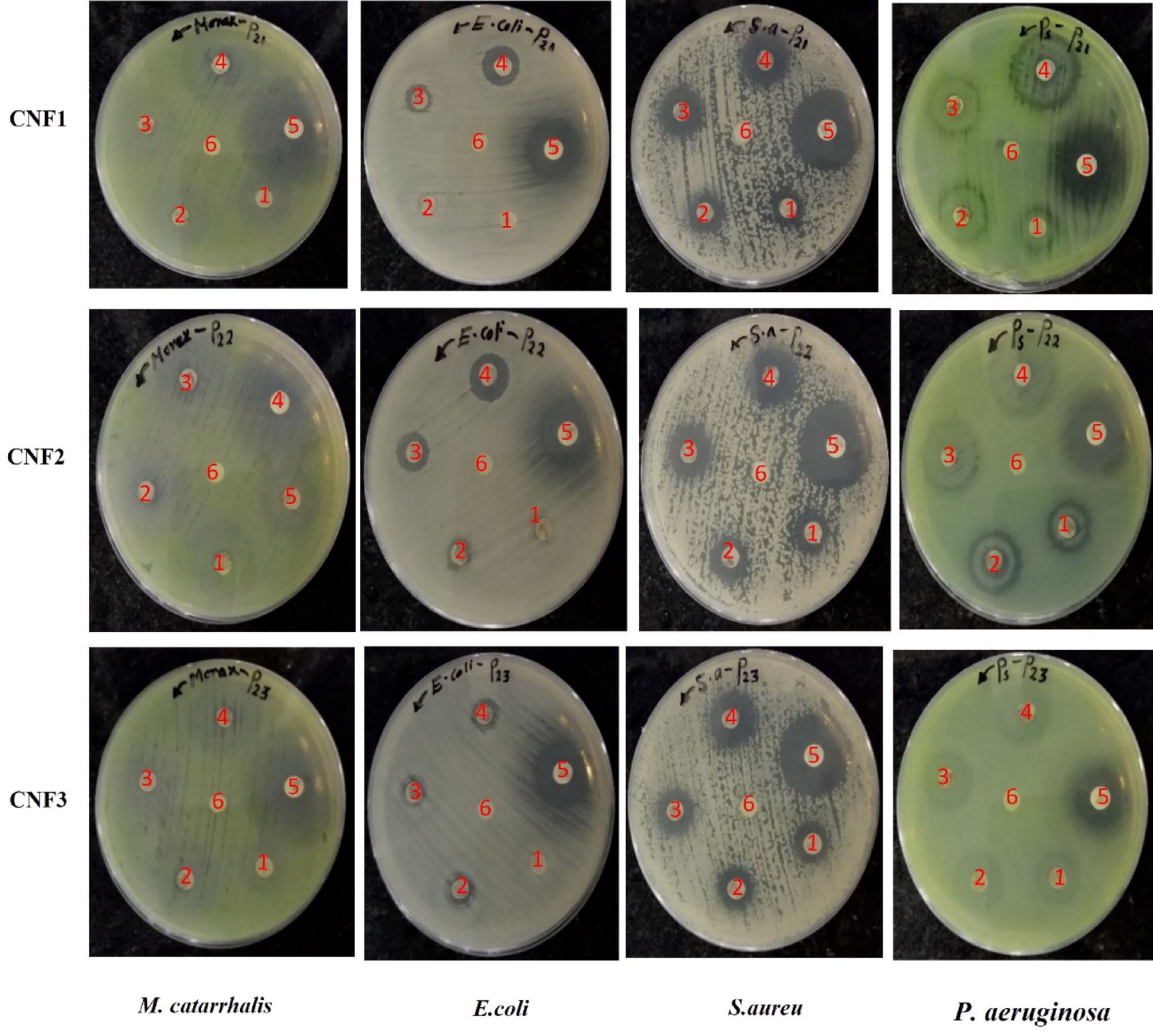
Antibacterial activity of CdO–NiO–Fe_2_O_3_ nanocomposites against bacteria: *(M. catarrhalis)*, (*E. coli),* (*S. aureus*) and *(P. aeruginosa)*. (1) 56.25, (2) 112.5, (3) 225 and (4) 450 μg/ml per disk of nanocomposites, (5) *Azithromycin antibiotics (positive control) and (6) distilled water (negative control).*

**Figure 7:**
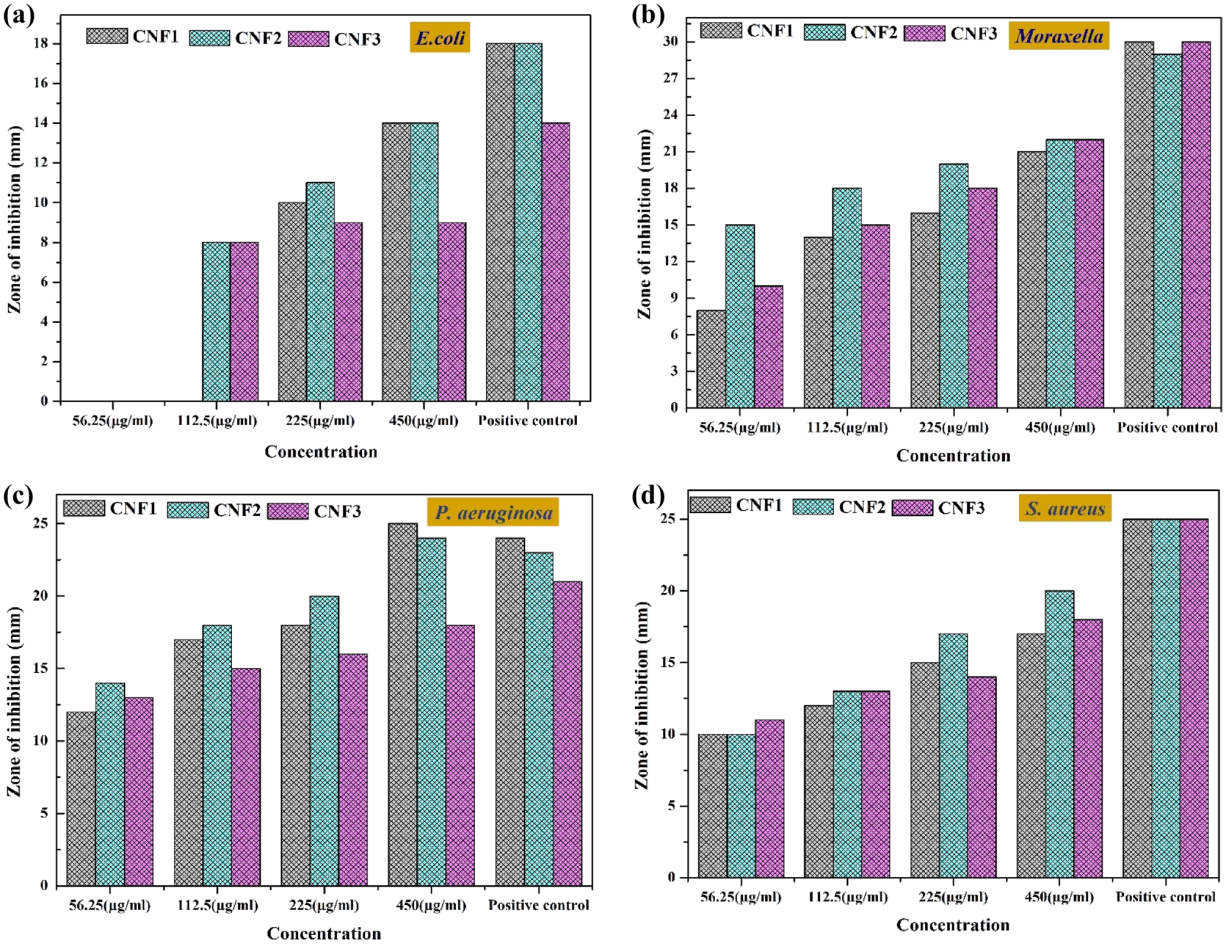
ZOI against (**a**) *E. coli*, (**b**) *Moraxella*, (**c**) *P. aeruginosa*, and (**d**) *S. aureus* bacterial strains in the presence of CNF1, CNF2 and CNF3 nanocomposites. The figure showed specialty of prepared nanocomposites, the CNF2 has a significant impact on bacteria compared to CNF1 and CNF3.

The antibacterial properties of the CdO–NiO–Fe_2_O_3_ nanocomposites were investigated against Gram-positive bacteria (*S. aureus*) and Gram-negative bacteria (*M. catarrhalis, E. coli*, and *P. aeruginosa*) (see Figs. 6, 7). The CdO-NiO-Fe_2_O_3_ nanocomposites are present at concentrations ranging from 56.25 to 450 µg/ml. The zone of inhibition (ZOI), which illustrates how the CdO–NiO–Fe_2_O_3_ nanocomposites affect bacterial growth, is shown in Figs. 6 and 7. The dramatic effects at 450 µg/ml were clearly visible. The ZOI of the CdO–NiO–Fe_2_O_3_ nanocomposites against the bacterial strains *E. coli, P-aeruginosa, S. aureus* and *M. catarrhalis* is 14, 25, 20 and 22 nm, respectively. In reality, the metal nanoparticles bind to the proteins and DNA of the pathogens by interacting with vital components such as the phosphorus (P) and sulphur (S) groups of bacterial DNA. As a result, bacterial DNA replication is destroyed^64^. One possible mechanism for the antibacterial effect is the production of free radicals. Through the damaged surface, the Cd^2+^, Ni^2+^ and Fe^3+^ ions in the nanocomposites penetrate the cell walls of the pathogens. Reactive oxygen species (ROS) are formed when ions are released from the nanoparticles. Superoxide radicals, hydroxyl radicals, singlet oxygen, and hydrogen peroxide are just some of the ROS components that have significant bactericidal activity^65–71^. The ZOI in this study compared with other studies is shown in Table 5.

**Table 5:** A comparison of antibacterial activity of CdO–NiO–Fe_2_O_3_ nanocomposites with some other metal oxide nanocomposites, metal oxides and compounds.

Nanoparticles (NPs)	Assessment method	Target bacteria				References
		*Escherichia coli*	*Staphylococcus aureus*	*Pseudomonas aeruginosa*	*Moraxella catarrhalis*	
CuO–MgO–ZnO	ZOI (mm), 1 mg/µL	26	19	22	–	^72^
CeO_2_–CuO–ZnO	ZOI (mm), 50 µg/µL	10	14	12	–	^73^
CdO–ZnO–MgO	ZOI (mm), 100 µg/mL	23	–	22	–	^35^
CdO–NiO–Fe_2_O_3_	ZOI (mm), 450 µg/ml	13	25	25	25	^67^
CdO–NiO–Fe_2_O_3_	ZOI (mm), 450 µg/ml	18	23	25	25	^68^
Ag–CuO	ZOI (mm), 20 µg/mL	2.1	0.75		–	^74^
AgI–CdO	ZOI (mm), 50 mg/ml	20	20	29	–	^75^
CS-MgO	MIC	11.9 µg/mL	9.8 µg/mL		–	^76^
CS-NiO	MIC	3.86 µg/mL	2.11 µg/mL		–	^76^
MgO	ZOI (mm), 20 µg/mL	6.8	7.2	–	–	^65^
NiO	ZOI (mm), 1 mg/mL	17.2	16.5	18.2	–	^77^
Polyoxotungstate (POT)	MIC	–	–	–	1 µg/mL	^78^
Aristolochia bracteolate	ZOI (mm)	–	–	–	12	^79^
CdO–NiO–Fe_2_O_3_	ZOI (mm), 450 µg/ml	14	25	20	22	Present work

## Conclusion

In conclusion, the preparation of CdO–NiO–Fe_2_O_3_ was successful, and its physical and antibacterial properties were studied. The molar ratio of Ni^2+^ and Fe^3+^ can affect the average crystallite size (D_av_), dislocation density (δ) and microstrain (ε). In particular, the results showed that the coupling of CdO with NiO and Fe_2_O_3_ improved the magnetic properties of CdO. At room temperature, the ferromagnetism of the CdO–NiO–Fe_2_O_3_ nanocomposites was enhanced, making them suitable for magnetic applications. According to the results, the grown nanocomposite showed high performance as antibacterial activity for various Gram-negative and positive bacteria, which could be a strong candidate for bacterial disinfection.

## Data Availability

The authors confirm that the data supporting the findings of this study are available within the article.
